# Supplementary material for: MorphoCluster: Efficient Annotation of Plankton Images by Clustering
Source: Sensors (Basel). 2020 May 28;20(11):3060. doi: 10.3390/s20113060 (PMC7308937; doi:10.3390/s20113060)
Supplement: Supplementary file 1 [file sensors-20-03060-s001.pdf]

Supplementary Materials: MorphoCluster: Efficient Annotation of Plankton images by Clustering

Simon-Martin Schröder [1,*], Rainer Kiko [2,3] and Reinhard Koch [1]

1. Label supercategories

This list accompanies Figure 6 and shows all labels contained in the colored supercategories.

artefact* (23, darkgray): artefact, artefact/badfocus, artefact/badfocus/badfocus_to_aggregate, artefact/badfocus/badfocus_to_oversegemented, artefact/badfocus/badfocus_to_oversegmented, artefact/badfocus/shrimp-badfocus_to_oversegmented, artefact/bubbles/bubbles-hexagonal, artefact/bubbles/bubbles-hexagonal_to_badfocus, artefact/bubbles/bubbles-two-halfmoons, artefact/bubbles/bubbles-two-stars, artefact/bubbles/bubbles-two-starts, artefact/cut, artefact/cut/cut-aggregates-jellies, artefact/cut/cut-aggregates-turbid, artefact/cut/cut-fibers_to_turbid, artefact/cut/cut-jellies, artefact/cut/cut-jellies-quadratic, artefact/cut/cut-tentacles, artefact/cut/tentacles-large-aggregates-cut, artefact/seafloor, artefact/turbid, artefact/turbid/turbid-fuzzy, artefact/turbid/turbid-w-objects

detritus/acantharia-remains_to_fiber* (1, darkgoldenrod): detritus/acantharia-remains_to_fiber

detritus/aggregate* (59, peru): detritus/aggregate/aggregate-angled-grey, detritus/aggregate/aggregate-angled-grey_to_crustacea, detritus/aggregate/aggregate-ball-dark, detritus/aggregate/aggregate-balls-fluffy-grey, detritus/aggregate/aggregate-balls-grey_to_aggregate-fluffy-grey, detritus/aggregate/aggregate-compact-fluffy-dark, detritus/aggregate/aggregate-compact-fluffy-grey, detritus/aggregate/aggregate-compact-fluffy_to_feces, detritus/aggregate/aggregate-compact-grey, detritus/aggregate/aggregate-compact-grey_to_rhizaria, detritus/aggregate/aggregate-compact-large-grey, detritus/aggregate/aggregate-compact-small-grey, detritus/aggregate/aggregate-dark-fluffy-ball, detritus/aggregate/aggregate-fiber-fluffy-dark, detritus/aggregate/aggregate-fiber-fluffy-grey, detritus/aggregate/aggregate-fluff-dark-edges, detritus/aggregate/aggregate-fluffy-dark, detritus/aggregate/aggregate-fluffy-dark-two-spots, detritus/aggregate/aggregate-fluffy-dark_to_feces, detritus/aggregate/aggregate-fluffy-dark_to_fiber-fluffy-dark, detritus/aggregate/aggregate-fluffy-grey, detritus/aggregate/aggregate-fluffy-large-compact-maybe-from-appendicularia, detritus/aggregate/aggregate-fluffy-light, detritus/aggregate/aggregate-fluffy-light-w-flota, detritus/aggregate/aggregate-fluffy-lightgrey, detritus/aggregate/aggregate-fluffy-lightgrey_to_feces, detritus/aggregate/aggregate-fluffy-loose-fiber_to_oversegmented, detritus/aggregate/aggregate-fluffy-loose-grey, detritus/aggregate/aggregate-fluffy-loose-grey_to_fluffy_fiber, detritus/aggregate/aggregate-fluffy-loose-lightgrey, detritus/aggregate/aggregate-fluffy-sinker_to_fiber-bundle, detritus/aggregate/aggregate-fluffy-very-faint_to_badfocus, detritus/aggregate/aggregate-fluffy-w-many-dark-spots, detritus/aggregate/aggregate-fluffy-w-two-dots, detritus/aggregate/aggregate-large-ball_to_globule, detritus/aggregate/aggregate-large-fluffy-and-appendicularia, detritus/aggregate/aggregate-large-marine-snow-w-black-parts_to_cut, detritus/aggregate/aggregate-long-fluffy-dark_to_fiber, detritus/aggregate/aggregate-rings-small-dark, detritus/aggregate/aggregate-sinker-large-fluffy, detritus/aggregate/aggregate-small-feathery_to_compact, detritus/aggregate/aggregate-small-fibers-grey_to_compact, detritus/aggregate/aggregate-small-grey-compact, detritus/aggregate/aggregate-thorny-grey, detritus/aggregate/aggregate-thorny-grey_to_crustacea, detritus/aggregate/aggregate-very-fluffy, detritus/aggregate/aggregate-very-fluffy-and-large, detritus/aggregate/aggregate-very-fluffy-loose-grey, detritus/aggregate/aggregate-very-large-fluffy, detritus/aggregate/aggregate-very-large-w-cut-tentacles, detritus/aggregate/aggregate-very-loose-grey, detritus/aggregate/aggregate-very-loose-w-dark-spots, detritus/aggregate/aggregate_dark_thorny_to_fiber, detritus/aggregate/aggregate_to_crustacea-decaying, detritus/aggregate/aggregate_to_solitary-black, detritus/aggregate/fluffy-grey, detritus/aggregate/jelly-like-remains-and-dark-spots, detritus/aggregate/small-aggregates-mixed-w-compact, detritus/aggregate/very-fluffy-possibly-discarded-appendicularia-houses

detritus/compact* (21, sienna): detritus/compact/compact-almond-grey_to_bubble, detritus/compact/compact-angled-dark, detritus/compact/compact-angled-dark_to_small-crustacea, detritus/compact/compact-dark, detritus/compact/compact-dark-thorny, detritus/compact/compact-dark-twins_to_bubble-stars, detritus/compact/compact-dark_to_aggregate-dark-fluffy, detritus/compact/compact-dark_to_crustacea, detritus/compact/compact-doubles-w-fluffy-surrounds, detritus/compact/compact-grey, detritus/compact/compact-grey-egg-form, detritus/compact/compact-grey-slightly-fluffy, detritus/compact/compact-grey-w-small-jellies_to_aggregate, detritus/compact/compact-light-grey_to_globule, detritus/compact/compact-round-black_to_solitary-black, detritus/compact/compact-small-dark_to_crustacea, detritus/compact/compact-small-round-grey, detritus/compact/compact-small-round-grey_to_solitary-black, detritus/compact/compact-thorny-dark_to_small-crustacea, detritus/compact/compact-w-fluffy-surrounds, detritus/compact/dark-compact-thorny_to_crustacea_small

detritus/crustacea-parts* (1, maroon): detritus/crustacea-parts

detritus/feces* (9, saddlebrown): detritus/feces/feces-bended-lengthy, detritus/feces/feces-dark-straight_to_trichodesmium-tuft, detritus/feces/feces-little-bended, detritus/feces/feces-little-fluffy, detritus/feces/feces-short-grey_to_trichodesmium-tuft,

detritus/feces/feces-small-grey, detritus/feces/feces-straight-faint-fluffy, detritus/feces/feces-straight-grey, detritus/feces/feces_to_trichodesmium-tuft

detritus/fiber* (31, goldenrod): detritus/fiber/fiber-bended-fluffy, detritus/fiber/fiber-bended-long-slightly-bundled, detritus/fiber/fiber-bended-ring-like, detritus/fiber/fiber-bended-thin, detritus/fiber/fiber-bended_to_tuft-sharp-ending, detritus/fiber/fiber-boundles-grey_to_aggregates-fluffy-grey, detritus/fiber/fiber-bundle, detritus/fiber/fiber-bundle-fluffy, detritus/fiber/fiber-bundle-grey, detritus/fiber/fiber-bundle-grey-small, detritus/fiber/fiber-bundle-large_to_aggregate, detritus/fiber/fiber-bundle-small, detritus/fiber/fiber-bundle-small-fragile, detritus/fiber/fiber-bundle-small_to_puff, detritus/fiber/fiber-fluffy-grey, detritus/fiber/fiber-fluffy-w-dark-spots, detritus/fiber/fiber-fluffy_to_feces, detritus/fiber/fiber-large-bundle, detritus/fiber/fiber-large-long-fluffy, detritus/fiber/fiber-long-slightly-bended-multiple, detritus/fiber/fiber-loops, detritus/fiber/fiber-medium-bended_to_feces, detritus/fiber/fiber-straight-w-knot, detritus/fiber/fiber-thin-w-dots, detritus/fiber/fiber-thin_to_chaetognatha, detritus/fiber/fiber_to_puff, detritus/fiber/fiber_to_rhizaria-spiky, detritus/fiber/fiber_to_small-crustacean, detritus/fiber/fiber_to_solitary-black, detritus/fiber/fluffy_fiber, detritus/fiber/fluffy_fiber/fluffy-fiber

detritus/rhizaria-remains_to_aggregate_small_fluffy* (1, tan): detritus/rhizaria-remains_to_aggregate_small_fluffy

metazoa/appendicularia* (2, mediumvioletred): metazoa/appendicularia/appendicularia-hous, metazoa/appendicularia/appendicularia-inner-house

metazoa/chaetognatha* (3, blueviolet): metazoa/chaetognatha, metazoa/chaetognatha/chaetognatha_to_badfocus, metazoa/chaetognatha/chaetognatha_to_cut

metazoa/crustacea* (41, red): metazoa/crustacea/amphipoda, metazoa/crustacea/amphipoda-like, metazoa/crustacea/copepoda, metazoa/crustacea/copepoda-dark_to_amphipoda, metazoa/crustacea/copepoda-like, metazoa/crustacea/copepoda-like_to_detritus_compact_angled_grey, metazoa/crustacea/copepoda-like_to_ostracoda-like, metazoa/crustacea/copepoda-to-detritus, metazoa/crustacea/copepoda/calanoida, metazoa/crustacea/copepoda/calanoida/dorsal-or-ventral, metazoa/crustacea/copepoda/calanoida/side-view, metazoa/crustacea/copepoda/calanoida/side-views, metazoa/crustacea/copepoda/copepoda-compact-dark, metazoa/crustacea/copepoda/copepoda-like, metazoa/crustacea/copepoda/copepoda-side-view_to_detritus_compact-grey, metazoa/crustacea/copepoda/copepoda-small-feathery, metazoa/crustacea/copepoda/harpacticoida, metazoa/crustacea/copepoda/mixed-view, metazoa/crustacea/copepoda/small-side-view, metazoa/crustacea/copepoda_to_badfocus, metazoa/crustacea/crustacea-like, metazoa/crustacea/crustacea_to_amphipoda-like, metazoa/crustacea/drop-like, metazoa/crustacea/drop-like_to_aggregate, metazoa/crustacea/drop-like_to_badfocus, metazoa/crustacea/drop-like_to_shrimp, metazoa/crustacea/ostracoda, metazoa/crustacea/pleuroncodes, metazoa/crustacea/shrimp, metazoa/crustacea/shrimp/shrimp-decaying-or-ill, metazoa/crustacea/shrimp/shrimp-front-view, metazoa/crustacea/shrimp/shrimp-head_to_shrimp-front-view, metazoa/crustacea/shrimp/shrimp-like, metazoa/crustacea/shrimp/shrimp-tails, metazoa/crustacea/shrimp/shrimp_to_oversegmented, metazoa/crustacea/shrimp_to_badfocus, metazoa/crustacea/shrimp_to_copepoda/drop_like, metazoa/crustacea/side-view, metazoa/crustacea/small_crustacea_to_feces, metazoa/crustacea/spider-like-amphipods, metazoa/crustacea/undefined

metazoa/drop-like* (1, salmon): metazoa/drop-like

metazoa/fish* (1, darkslateblue): metazoa/fish

metazoa/jellies* (14, darkcyan): metazoa/jellies/cnidaria/bitentaculata, metazoa/jellies/cnidaria/fringed-jellies-w-dot, metazoa/jellies/cnidaria/jellies-large-medusa, metazoa/jellies/cnidaria/jellies-w-cross, metazoa/jellies/cnidaria/jellies-w-dot-and-edges, metazoa/jellies/cnidaria/jellies-w-dots_to_badfocus, metazoa/jellies/cnidaria/jellies-w-stripes, metazoa/jellies/cnidaria/jelly-small-rings_to_badcocus, metazoa/jellies/cnidaria/medusa-compact, metazoa/jellies/cnidaria/medusa-large_to_cut, metazoa/jellies/cnidaria/round-jellies-w-dot, metazoa/jellies/cnidaria/small-jellies-w-dot, metazoa/jellies/ctenophora/beroe-type, metazoa/jellies/ctenophora/top-view

metazoa/mollusca* (4, indigo): metazoa/mollusca/snail, metazoa/mollusca/veliger, metazoa/mollusca/veliger/veliger-straight-arms, metazoa/mollusca/veliger/veliger_to_solitary-black

metazoa/polychaeta* (6, magenta): metazoa/polychaeta, metazoa/polychaeta/flota, metazoa/polychaeta/long-bended-worms, metazoa/polychaeta/poeobius, metazoa/polychaeta/short-bended-worms-feathery, metazoa/polychaeta/worms_to_badfocus

metazoa/salpida-larvae* (1, pink): metazoa/salpida-larvae

mix* (5, lightgray): mix/crustacea_to_badfocus, mix/detritus_to_chaetognatha, mix/detritus_to_crustacea, mix/fiber_tentacles, mix/mix-of-different-grey-items

rhizaria/* (14, aqua): rhizaria/almond-eye, rhizaria/collodaria/colonial, rhizaria/double-lobes, rhizaria/eight-armed, rhizaria/rhizaria-mix, rhizaria/rhizaria-small_to_compact, rhizaria/rhizaria_to_detritus, rhizaria/six-lobes, rhizaria/sphere-eye-w-spikes, rhizaria/triangular-eye-w-spikes, rhizaria/triangular-sphere, rhizaria/triangular-sphere_to_sphere-legs, rhizaria/unknown-four-lobes-w-spikes, rhizaria/unknown-quadruple-colony

rhizaria/acantharia* (3, lightblue): rhizaria/acantharia, rhizaria/acantharia/acantharia-small_to_solitary-black, rhizaria/acantharia_to_spiky

rhizaria/foraminifera* (5, skyblue): rhizaria/foraminifera/foraminifera-tight, rhizaria/foraminifera/foraminifera_to_foraminifera-cut, rhizaria/foraminifera_to_fiber-bundle-fluffy, rhizaria/foraminifera_to_sphere-legs, rhizaria/foraminifera_to_spiky

rhizaria/globule* (3, deepskyblue): rhizaria/globule/globule_to_badfocus, rhizaria/globule/globule_to_sphere_thorn, rhizaria/globule/small-globule
rhizaria/solitary-black* (11, cornflowerblue): rhizaria/solitary-black, rhizaria/solitary-black-faint, rhizaria/solitary-black-like, rhizaria/solitary-black-like_to_acantharia, rhizaria/solitary-black-like_to_rhizaria, rhizaria/solitary-black/solitary-black-large, rhizaria/solitary-black/solitary-black-like, rhizaria/solitary-black/solitary-black-small, rhizaria/solitary-black/solitary-black-small-w-grey-surrounds, rhizaria/solitary-black/solitary-black_to_sphere_eye, rhizaria/solitary-black_to_puff
rhizaria/sphere-thorn* (6, dodgerblue): rhizaria/sphere-thorn, rhizaria/sphere-thorn/sphere-thorn-doubles, rhizaria/sphere-thorn/sphere-thorn_to_badfocus, rhizaria/sphere-thorn/sphere_thorn/sphere-thorn_to_badfocus, rhizaria/sphere-thorn_to_legs, rhizaria/sphere-thorns-w-balls
trichodesmium/puff* (6, greenyellow): trichodesmium/puff, trichodesmium/puff/puff-large, trichodesmium/puff/puff-large-small, trichodesmium/puff/puff-large_to_fiber-bundle, trichodesmium/puff/puff-medium, trichodesmium/puff/puff-small
trichodesmium/tuft* (5, limegreen): trichodesmium/tuft/feathery-ending, trichodesmium/tuft/feathery-sharp-ending, trichodesmium/tuft/sharp-ending, trichodesmium/tuft/trichodesmium-tuft-dark_to_feces, trichodesmium/tuft/tuft-grey-irregular
unknown* (15, gainsboro): unknown, unknown/ball-w-tentacles, unknown/eggs-maybe, unknown/eye-slit, unknown/fiber_w_growth_in_middle, unknown/half-moon-w-dot, unknown/half-moon-w-dot_to_badfocus, unknown/halfmoon-w-dot, unknown/jelly, unknown/long-even, unknown/ovoid-w-dark-center-line, unknown/ovoid-w-dot, unknown/unkown/appendicularia_body_to_harpacticoid, unknown/unkown/small-rings-w-dots, unknown/weirdo

2. Corresponding labels

This list accompanies Figure 7 and contains the corresponding names in $\mathcal{L}_0$ and $\mathcal{L}_{MC}$. The initial class names are in the same order as in the figure. Manually established correspondences are printed in bold.

Annelida_Polychaeta (0): *n/a*
Crustacea_leg (0): *n/a*
Diplostraca_Cladocera (0): *n/a*
Euopisthobranchia_Thecosomata (0): *n/a*
Mollusca_Cephalopoda (0): *n/a*
Pyrosomatida_Pyrosoma (0): *n/a*
Solmundella_Solmundella bitentaculata (0): *n/a*
detritus_light (0): *n/a*
othertocheck_darksphere (0): *n/a*
temporary_t009 (0): *n/a*
Appendicularia_body (1): aggregate/aggregate-balls-grey_to_aggregate-fluffy-grey
Arthropoda_Crustacea (1): crustacea/spider-like-amphipods
Collodaria_solitaryfuzzy (1): rhizaria/solitary-black-like_to_rhizaria
Euopisthobranchia_Gymnosomata (1): fiber/fiber-boundles-grey_to_aggregates-fluffy-grey
Munididae_Pleuroncodes (1): **crustacea/pleuroncodes**
Terebellida_Flota (1): **polychaeta/flota**
Thaliacea_Salpida (1): cut/cut-aggregates-jellies
Trachylina_Narcomedusae (1): cnidaria/medusa-large_to_cut
temporary_t002 (1): **rhizaria/eight-armed**
temporary_t003 (1): **rhizaria/six-lobes**
temporary_t005 (1): **rhizaria/sphere-thorns-w-balls**
temporary_t006 (1): **metazoa/salpida-larvae**
temporary_t015 (1): aggregate/aggregate-fluffy-dark_to_fiber-fluffy-dark
Appendicularia_house (2): aggregate/aggregate-large-fluffy-and-appendicularia, aggregate/very-fluffy-possibly-discarded-appendicularia-houses
Hydroidolina_Siphonophorae (2): artefact/cut, aggregate/aggregate-very-large-w-cut-tentacles
Metazoa_Ctenophora (2): cut/cut-aggregates-turbid, ctenophora/beroe-type
Vertebrata_Gnathostomata (2): **metazoa/fish**, chaetognatha/chaetognatha_to_cut
temporary_t004 (2): **rhizaria/triangular-sphere**, rhizaria/triangular-eye-w-spikes
temporary_t010 (2): aggregate/aggregate_dark_thorny_to_fiber, feces/feces-little-fluffy
temporary_t012 (2): aggregate/aggregate-fluffy-lightgrey, feces/feces-straight-faint-fluffy
Aulacanthidae_Aulacantha (3): **rhizaria/sphere-thorn**, rhizaria/rhizaria-mix, rhizaria/sphere-thorn_to_legs
Metazoa_Mollusca (3): compact/compact-dark_to_aggregate-dark-fluffy, crustacea/crustacea-like, rhizaria/double-lobes
Phaeosphaerida_Aulosphaeridae (3): **rhizaria/sphere-eye-w-spikes**, solitary-black/solitary-black-small-w-grey-surrounds, sphere-thorn/sphere-thorn_to_badfocus
Terebellida_Poeobius (3): **polychaeta/poeobius**, compact/compact-doubles-w-fluffy-surrounds, fiber/fiber-bundle
Tunicata_Appendicularia (3): aggregate/aggregate-large-marine-snow-w-black-parts_to_cut, aggregate/aggregate-very-fluffy-and-large, appendicularia/appendicularia-hous
artefact_turbid (3): **artefact/turbid**, turbid/turbid-fuzzy, turbid/turbid-w-objects

detritus_compact (3): aggregate/aggregate-fluff-dark-edges, compact/compact-dark, compact/compact-small-dark_to_crustacea
temporary_t001 (3): fiber/fiber-loops, rhizaria/almond-eye, unknown/halfmoon-w-dot
Hydrozoa_Cnidaria (4): aggregate/aggregate-very-fluffy, cnidaria/fringed-jellies-w-dot, cnidaria/jellies-w-cross, cnidaria/jellies-w-stripes
Retaria_Foraminifera (4): cut/cut-tentacles, foraminifera/foraminifera-tight, foraminifera/foraminifera_to_foraminifera-cut, rhizaria/foraminifera_to_spiky
Metazoa_Chaetognatha (5): metazoa/chaetognatha, fiber/fiber-fluffy-grey, fiber/fiber-thin_to_chaetognatha, chaetognatha/chaetognatha_to_badfocus, mix/fiber_tentacles
Mollusca_veliger (5): mollusca/veliger, aggregate/aggregate_to_solitary-black, aggregate/small-aggregates-mixed-w-compact, detritus/rhizaria-remains_to_aggregate_small_fluffy, veliger/veliger-straight-arms
Oligostraca_Ostracoda (5): crustacea/ostracoda, aggregate/aggregate-angled-grey, compact/compact-dark-thorny, copepoda/copepoda-side-view_to_detritus-compact-grey, mix/mix-of-different-grey-items
Retaria_Acantharea (5): rhizaria/acantharia, aggregate/aggregate-compact-large-grey, fiber/fiber_to_solitary-black, rhizaria/acantharia_to_spiky, tuft/tuft-grey-irregular
detritus_ovoid (5): aggregate/aggregate-compact-grey, aggregate/aggregate-rings-small-dark, compact/compact-grey-egg-form, compact/compact-grey-slightly-fluffy, unknown/eye-slit
fiber_fluffy (5): aggregate/aggregate-compact-fluffy_to_feces, aggregate/aggregate-fluffy-w-two-dots, aggregate/aggregate-very-loose-w-dark-spots, fiber/fiber-fluffy-w-dark-spots, unkown/appendicularia_body_to_harpacticoid
fluffy_light (5): aggregate/aggregate-fluffy-loose-grey_to_fluffy_fiber, aggregate/aggregate-fluffy-loose-lightgrey, aggregate/aggregate-very-fluffy-loose-grey, mix/detritus_to_crustacea, unknown/ovoid-w-dot
temporary_t011 (5): aggregate/aggregate-fiber-fluffy-dark, aggregate/aggregate-fiber-fluffy-grey, aggregate/aggregate-fluffy-dark, aggregate/aggregate-fluffy-lightgrey_to_feces, aggregate/aggregate-thorny-grey_to_crustacea
Collodaria_collonial (6): collodaria/colonial, aggregate/aggregate-fluffy-large-compact-maybe-from-appendicularia, aggregate/aggregate-large-ball_to_globule, aggregate/aggregate-very-loose-grey, sphere-thorn/sphere-thorn-doubles, unkown/small-rings-w-dots
Collodaria_solitaryblack (6): rhizaria/solitary-black, compact/compact-round-black_to_solitary-black, rhizaria/rhizaria_to_detritus, rhizaria/solitary-black-like_to_acantharia, solitary-black/solitary-black-large, solitary-black/solitary-black_to_sphere_eye
Collodaria_solitarygrey (6): aggregate/aggregate-ball-dark, aggregate/aggregate-fluffy-w-many-dark-spots, compact/compact-small-round-grey_to_solitary-black, acantharia/acantharia-small_to_solitary-black, rhizaria/rhizaria-small_to_compact, rhizaria/solitary-black-faint
Metazoa_Cnidaria (6): cut/cut-jellies, aggregate/aggregate-balls-fluffy-grey, cnidaria/bitentaculata, cnidaria/jellies-large-medusa, cnidaria/jellies-w-dot-and-edges, cnidaria/round-jellies-w-dot
Trichodesmium_tuff (6): feces/feces-short-grey_to_trichodesmium-tuft, fiber/fiber-bundle-grey-small, puff/puff-large_to_fiber-bundle, tuft/feathery-ending, tuft/feathery-sharp-ending, tuft/sharp-ending
Cnidaria_Hydrozoa (7): aggregate/aggregate-compact-fluffy-grey, aggregate/aggregate-fluffy-light, compact/compact-grey-w-small-jellies_to_aggregate, fiber/fiber-bended-ring-like, cnidaria/jelly-small-rings_to_badcocus, cnidaria/small-jellies-w-dot, unknown
Phaeodaria_leg (7): rhizaria/foraminifera_to_sphere-legs, detritus/acantharia-remains_to_fiber, fiber/fiber-bundle-small, fiber/fiber_to_rhizaria-spiky, rhizaria/foraminifera_to_fiber-bundle-fluffy, rhizaria/solitary-black-like, rhizaria/triangular-sphere_to_sphere-legs
Collodaria_solitaryglobule (8): aggregate/aggregate-compact-fluffy-dark, aggregate/aggregate-compact-grey_to_rhizaria, aggregate/aggregate-compact-small-grey, compact/compact-light-grey_to_globule, cnidaria/jellies-w-dots_to_badfocus, globule/globule_to_badfocus, globule/globule_to_sphere_thorn, globule/small-globule
artefact_badfocus (8): artefact/badfocus, badfocus/badfocus_to_aggregate, badfocus/badfocus_to_oversegmented, bubbles/bubbles-hexagonal_to_badfocus, cut/cut-jellies-quadratic, aggregate/aggregate-fluffy-loose-fiber_to_oversegmented, aggregate/aggregate-fluffy-very-faint_to_badfocus, fiber/fiber-fluffy_to_feces
artefact_bubble (8): bubbles/bubbles-hexagonal, bubbles/bubbles-two-halfmoons, bubbles/bubbles-two-stars, compact/compact-almond-grey_to_bubble, compact/compact-dark-twins_to_bubble-stars, compact/compact-grey, compact/compact-w-fluffy-surrounds, fiber/fiber-bended_to_tuft-sharp-ending
not-living_feces (8): compact/compact-angled-dark, feces/feces-dark-straight_to_trichodesmium-tuft, feces/feces-little-bended, feces/feces-small-grey, feces/feces-straight-grey, feces/feces_to_trichodesmium-tuft, tuft/trichodesmium-tuft-dark_to_feces, unknown/long-even
Metazoa_Annelida (9): aggregate/aggregate-fluffy-light-w-flota, aggregate/aggregate-long-fluffy-dark_to_fiber, fiber/fiber-bended-long-slightly-bundled, fiber/fiber-bundle-large_to_aggregate, fiber/fiber-large-long-fluffy, metazoa/polychaeta, polychaeta/long-bended-worms, polychaeta/worms_to_badfocus, mix/detritus_to_chaetognatha
detritus_fiber (10): cut/cut-fibers_to_turbid, aggregate/aggregate-fluffy-sinker_to_fiber-bundle, feces/feces-bended-lengthy, fiber/fiber-bended-fluffy, fiber/fiber-bended-thin, fiber/fiber-long-slightly-bended-multiple, fiber/fiber-medium-bended_to_feces, fiber/fiber-straight-w-knot, fiber/fiber-thin-w-dots, unknown/half-moon-w-dot_to_badfocus

fluffy_dark (12): aggregate/aggregate-angled-grey_to_crustacea, aggregate/aggregate-dark-fluffy-ball, aggregate/aggregate-fluffy-dark-two-spots, aggregate/aggregate-fluffy-dark_to_feces, aggregate/aggregate-fluffy-grey, aggregate/aggregate-fluffy-loose-grey, aggregate/aggregate-small-fibers-grey_to_compact, aggregate/jelly-like-remains-and-dark-spots, crustacea/copepoda-dark_to_amphipoda, copepoda/copepoda-compact-dark, crustacea/undefined, ctenophora/top-view

Trichodesmium_puff (13): **trichodesmium/puff**, aggregate/aggregate-small-feathery_to_compact, compact/compact-angled-dark_to_small-crustacea, compact/compact-small-round-grey, fiber/fiber-bundle-grey, fiber/fiber-bundle-small_to_puff, fiber/fiber_to_puff, solitary-black/solitary-black-small, rhizaria/solitary-black_to_puff, puff/puff-large, puff/puff-large-small, puff/puff-medium, puff/puff-small

Maxillopoda_Copepoda (19): **crustacea/copepoda**, aggregate/aggregate-thorny-grey, compact/compact-thorny-dark_to_small-crustacea, fiber/fiber_to_small-crustacean, crustacea/amphipoda-like, crustacea/copepoda-like, crustacea/copepoda-like_to_detritus_compact_angled_grey, crustacea/copepoda-like_to_ostracoda-like, crustacea/copepoda-to-detritus, copepoda/calanoida, calanoida/dorsal-or-ventral, calanoida/side-view, copepoda/copepoda-small-feathery, copepoda/mixed-view, copepoda/small-side-view, crustacea/drop-like_to_aggregate, crustacea/small_crustacea_to_feces, unknown/ball-w-tentacles, unknown/fiber_w_growth_in_middle

Malacostraca_Eumalacostraca (25): **crustacea/shrimp**, badfocus/shrimp-badfocus_to_oversegmented, aggregate/aggregate-sinker-large-fluffy, aggregate/aggregate-very-large-fluffy, aggregate/aggregate_to_crustacea-decaying, detritus/crustacea-parts, fiber/fiber-large-bundle, fiber/fluffy_fiber, crustacea/amphipoda, copepoda/harpacticoida, crustacea/copepoda_to_badfocus, crustacea/crustacea_to_amphipoda-like, crustacea/drop-like, crustacea/drop-like_to_badfocus, crustacea/drop-like_to_shrimp, shrimp/shrimp-decaying-or-ill, shrimp/shrimp-front-view, shrimp/shrimp-like, shrimp/shrimp-tails, shrimp/shrimp_to_oversegmented, crustacea/shrimp_to_badfocus, shrimp_to_copepoda/drop_like, crustacea/side-view, metazoa/drop-like, mix/crustacea_to_badfocus
